# A New Bioactive Metabolite Isolated from the Red Sea Marine Sponge *Hyrtios erectus*

**DOI:** 10.3390/molecules21010082

**Published:** 2016-01-15

**Authors:** Sameh S. Elhady, Ali M. El-Halawany, Abdulrahman M. Alahdal, Hashim A. Hassanean, Safwat A. Ahmed

**Affiliations:** 1 Department of Pharmacognosy, Faculty of Pharmacy, Suez Canal University, Ismailia 41522, Egypt; ssahmed@kau.edu.sa (S.S.E.) hashem_omar@pharm.suez.edu.eg (H.A.H.); 2 Department of Natural Products and Alternative Medicine, Faculty of Pharmacy, King Abdulaziz University, Jeddah 21589, Saudi Arabia; ahalawany2003@yahoo.com; 3 Pharmacognosy Department, Faculty of Pharmacy, Cairo University, Kasr el-Aini Street, Cairo 11562, Egypt; 4 Department of Clinical Pharmacy, Faculty of Pharmacy, King Abdulaziz University, Jeddah 21589, Saudi Arabia; aalahdal2@hotmail.com

**Keywords:** Red Sea sponge, *Hyrtios erectus*, 24-methoxypetrosaspongia C, scalarane sesterterpenes, cancer cell lines, growth inhibitory activity

## Abstract

Chemical investigation of the lipophilic fraction of *Hyrtios erectus*, a Red Sea sponge, yielded a new pentacyclic nitrogen-containing scalarane; 24-methoxypetrosaspongia C (**1**), together with the previously reported scalaranes sesterstatin 3 (**2**), 12-deacetyl-12-*epi*-scalaradial (**3**) and 12-deacetyl-12,18-di-*epi*-scalaradial (**4**). The compounds were identified using HRESIMS, 1D and 2D NMR experiments. The isolated compounds showed growth inhibitory activity against hepatocellular carcinoma (HepG2), colorectal carcinoma (HCT-116) and breast adenocarcinoma cells (MCF-7).

## 1. Introduction

Marine organisms have always been an attractive source of natural products with novel and exotic structures and useful biological activities [1,2]. Marine sponges of the order Dictyoceratida have yielded many types of scalarane sesterterpenes [1]. Scalarane sesterterpenoids possess a variety of biological activities, including anti-cancer [3,4,5,6,7,8,9,10], antimicrobial [11,12], antifeedant [13], ichthyotoxic [14], anti-inflammatory [15,16] and platelet-aggregation inhibitory effects [17]. A wide variety of structurally diverse substances with potentially useful biological activities have been isolated from the *Hyrtios* genus, including terpenoids [3,4,5,6,7,8,9,18,19], macrolides [4,20,21,22], and tryptamine-derived alkaloids [19,23,24,25].

In our pursuit of natural drug leads from Red Sea marine sponges, the chemical investigation of the antiproliferative organic extract of the Red Sea sponge *Hyrtios erectus*, family Thorectidae was carried out. The study resulted in the identification of four scalarane sesterterpenes including the new compound 24-methoxypetrosaspongia C (**1**), and the previously reported scalaranes sesterstatin 3 (**2**) [4], 12-deacetyl-12-*epi*-scalaradial (**3**) [16] and 12-deacetyl-12,18-di-*epi*-scalaradial (**4**) [13]. Herein, the purification, structure determination and growth inhibitory effects of compounds **1**–**4** will be discussed.

## 2. Results and Discussion

### 2.1. Purification of Compounds ***1**–**4***

The lipophilic extract of the marine sponge, *H. erectus* was subjected to a series of chromatographic separations using silica gel column chromatography, followed by HPLC purification. The separation procedures resulted in the isolation of the new compound 24-methoxy-petrosaspongia C (**1**), along with three previously reported compounds: sesterstatin 3 (**2**), 12-deacetyl-12-*epi*-scalaradial (**3**) and 12-deacetyl-12,18-di-*epi*-scalaradial (**4**).

### 2.2. Structure Elucidation of Compound ***1**–**4***

**Figure 1:**
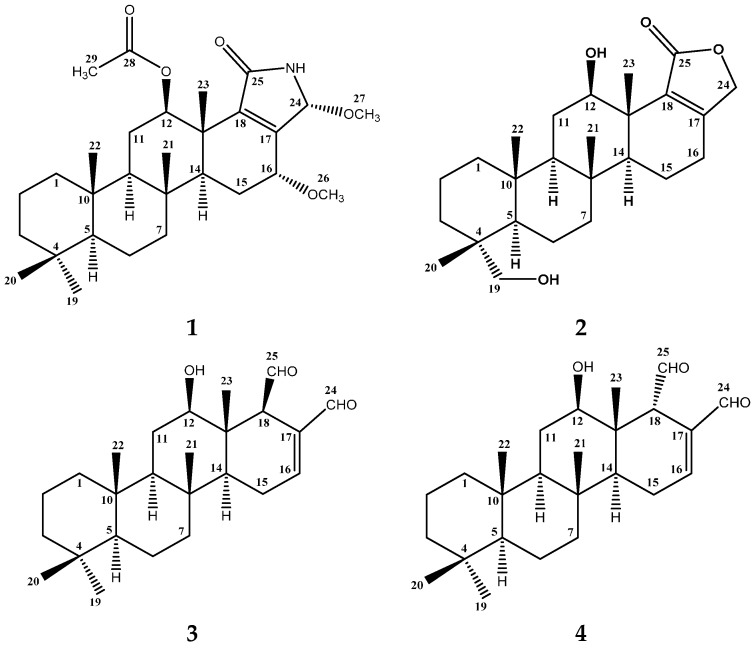
Structure of isolated scalarane sesterterpenes **1**–**4**.

**Figure 2:**
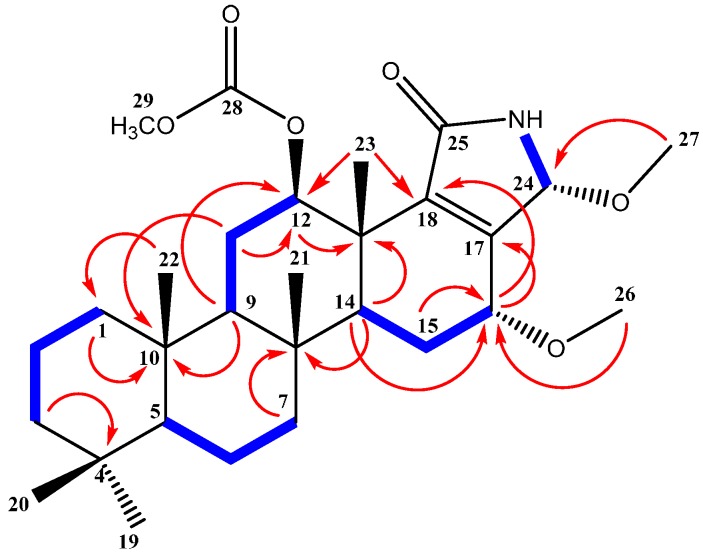
HMBC correlations (arrows) and COSY connectivities (bold bonds) of compound **1**.

Compound **1** (Figure 1) was isolated and purified as an amorphous solid. The molecular formula C_29_H_45_NO_5_ was established from the positive HRESIMS (high-resolution electrospray ionization mass spectrometry) pseudomolecular ion peak at *m*/*z* 488.3374 [M + H]^+^. The ^1^H-NMR spectrum of compound **1** (Table 1) displayed resonances for 45 protons, including five singlets belonging to five methyl groups (δ_H_ 0.80, 0.82, 0.84, 0.91 and 1.20), two methoxyls, (δ_H_ 3.20 and 3.39), one acetyl methyl (δ_H_ 2.12) seven methylenes, six aliphatic methines, and an exchangeable broad signal at δ_H_ 5.78 for a NH moiety (Supplementary Materials, Figure S1). The ^13^C-NMR spectrum (Table 1) showed signals for 29 carbons, including eight methyls, seven methylenes, six methines , and eight quaternary carbons (Supplementary Materials, Figure S2). Analysis of the ^1^H,^1^H-COSY and the HSQC NMR experiments led to the assembly of the following structural fragments: C-1 to C-3; C-5 to C-7; C-9 to C-12 with an acetoxy group at C-12; C-14 to C-16 with methoxy group at C-16 and C-16 to C-24 with methoxy group at C-24. These fragments allowed identifying a 12-acetoxy-16-methoxyscalarane skeleton (Figure 2) based on the correlations of H-12 and H-16 with neighboring protons and carbons in the COSY and HMBC (Supplementary Materials, Figures S3–S5). The C-17/C-18 double bond was inferred by heteronuclear long range correlations between H_3_-23 at δ_H_ 1.20 and the quaternary olefinic carbon at δ_C_ 144.7 (C-18) and between H-16 at δ_H_ 3.85 and the olefinic carbon at δ_C_ 151.1 (C-17). Furthermore, the ^13^C chemical shifts of C-17 and C-18 indicated the location of the amide carbonyl at C-25 [26].

**Table 1:** NMR data and HMBC correlations of compound **1** (CDCl_3_).

Position	δ_C_	δ_H_ (m, *J* in Hz)	HMBC (H→C) ^a^
1	39.6, CH_2_	1.62, 0.83 (m)	C-10
2	18.4, CH_2_	1.59, 1.41 (m)	C-4, C-10
3	42.0 CH_2_	1.35, 1.11 (m)	C-4
4	33.2 qC		
5	56.3 CH	0.80 (m)	C-4
6	18.2 CH_2_	1.58, 1.42 (m)	
7	41.3 CH_2_	1.81, 0.95 (m)	C-8
8	36.7 qC		
9	57.5 CH	1.01 (m)	C-10, C-12
10	37.3 qC		
11	24.8 CH2	1.77, 1.54 (m)	C-10, C-12
12	75.3 CH	4.95 (dd, 10.8, 4.8)	C-11, C-13, C-18, C-23, C-28
13	42.1 qC		
14	50.0 CH	1.44 (m)	C-8, C-9, C-13, C-16, C-18
15	21.3 CH_2_	2.01, 1.55 (m)	
16	69.8 CH	3.85 (dd, 4.2, 1.2)	C-17, C-18, C-26, C-24
17	151.1 qC		
18	144.7 qC		
19	21.3 CH_3_	0.80 (s)	C-4
20	33.2 CH_3_	0.84 (s)	C-4
21	17.3 CH_3_	0.91 (s)	C-7, C-8, C-9, C-14
22	15.7 CH_3_	0.82 (s)	C-1, C-5, C-9, C-10
23	15.9 CH_3_	1.20 (s)	C-12, C-13, C-18, C-14
24	82.3 CH	5.3 (s)	C-17, C-18, C-25, C-27
25	170.5 qC		
26	57.1 OCH_3_	3.39 (s)	C-16
27	52.1 OCH_3_	3.20 (s)	C-24
28	171.4 qC		
29	21.9 CH_3_	2.12 (s)	C-28
NH		5.78 (br s)	C-17, C-18

^a^ HMBC correlations are from proton(s) stated to the indicated carbons.

**Figure 3:**
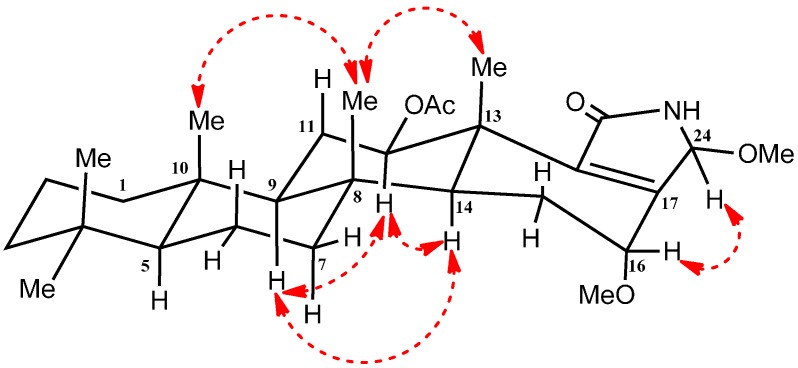
Important NOESY NMR correlations of compound **1**.

The relative configurations at C-12, C-16 and C-24 were detected and confirmed by their coupling constants and NOESY correlations (Supplementary Materials, Figure S6). The diaxial coupling of H-12 (δ_H_ 4.95; dd, *J* = 10.8 Hz) with H-11 indicates its α orientation. Moreover, NEOSY correlation with the α oriented H-9 and H-14 confirms α orientation of H-12 (Figure 3). While, lack of the diaxial coupling of H-16 (δ_H_ 3.85; dd, *J* = 4.2, 1.2 Hz) with H-15 indicates the equatorial orientation of H-16 (Figure 3). Finally, NOESY correlations between H-16 and H-24 revealed its β orientation (Figure 3). Therefore, compound **1** was identified as 24-methoxypetrosaspongia C.

Compound **1** represents a further example of scalaranes containing nitrogen, a group which which includes petrosaspongiolactams A-C [26], hyatelactam [27], and the pyrrole-terpenes molliorins A [28], molliorins B [29], and molliorins C [30].

The known compounds **2**–**4** (Figure 1) were identified by extensive study of their spectral data, including ESIMS, 1D and 2D NMR data, as well as by comparison with the available data in the literature. Thus, the compounds were identified as sesterstatin 3 (**2**) [4], 12-deacetyl-12-*epi*-scalaradial (**3**) [16] and 12-deacetyl-12,18-di-*epi*-scalaradial (**4**) [13].

### 2.3. Biological Activity of the Isolated Compounds

The growth inhibitory effects of compounds **1**–**4** (Table 2) against breast adenocarcinoma (MCF-7), hepatocellular carcinoma (HepG2) and colorectal carcinoma cells (HCT-116) were evaluated using a logarithmic best fit equation (E_max_ model Equation). Compound **4** was the most potent against all tested cell lines with IC_50_ 3.3, 1.7 and 3.4 µM in MCF-7, HepG2, and HCT-116 cell lines, respectively. Both compounds **1** and **3** showed intermediate activities with IC_50_ 55.4, 25.4 and 26.5 µM for compound **1** and 36.0, 23.4 and 27.1 µM for compound **3** against MCF-7, HepG2, and HCT-116 cell lines, respectively. On other hand, compound **2** showed no growth inhibitory effects against all tested cell lines.

**Table 2:** Growth-inhibitory activity of compounds **1**–**4** (*in vitro* IC_50_ (µM) growth-inhibitory values) against three human solid tumor cell lines.

Cell Type	Cell Line	Doxorubicin ^a^	1	2	3	4
Breast	MCF-7	0.41	55.4	>100	36.0	3.3
Hepatocellular	HepG2	0.85	25.4	>100	23.4	1.7
Colorectal	HCT-116	0.11	26.5	>100	27.1	3.4

^a^ positive cytotoxic control.

Furthermore, the morphological changes induced by compounds **1**, **3** and **4** were carried out against HCT-116 cells using computer-assisted phase-contrast microscopy [31,32,33]. In addition, the effect of compounds **1**, **3** and **4** on cell membrane integrity was quantified using a lactate dehydrogenase leakage assay (LDH leakage assay).

**Figure 4:**
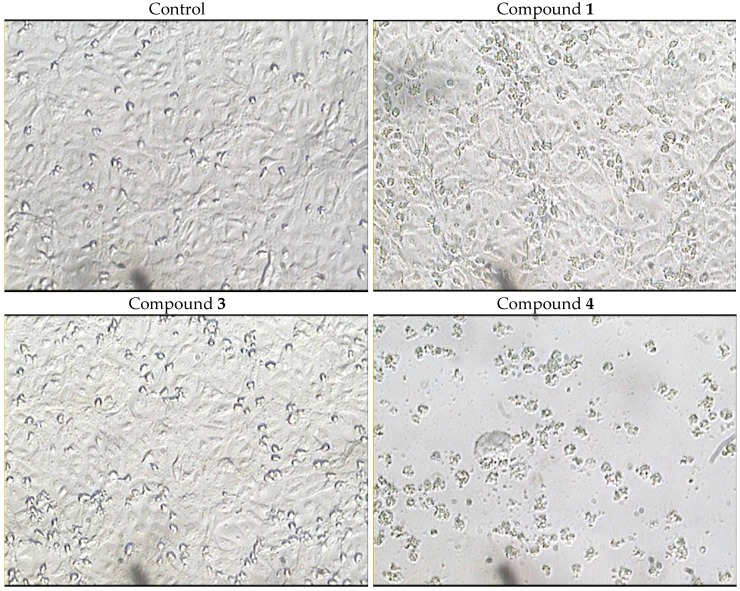
Morphological abnormalities induced by treating HCT-116 cells with compounds **1**, **3** and **4** (10 µM) for 72 h.

**Figure 5:**
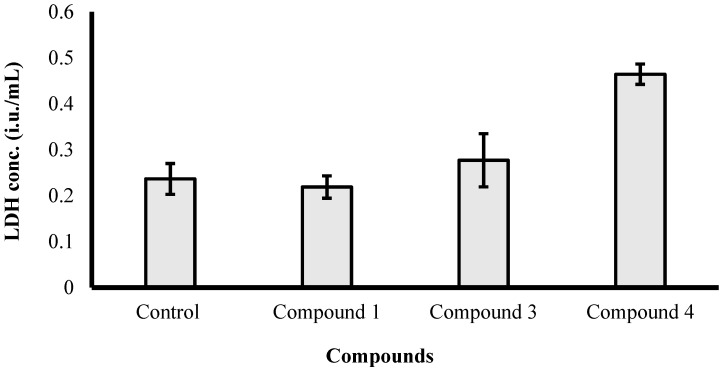
Assessing lactate dehydrogenase (LDH) leakage from HCT-116 after exposure to 10 µM of compounds **1**, **3** and **4** for 72 h.

The results illustrated in Figure 4 reveal that among the three active compounds against HCT-116 cells (Table 2), compound **4** induced apparent morphological changes suggesting a cytotoxic effect (cell killing). These morphological abnormalities attributed to cell exposure to compound **4** were not observed after treatment with the other two compounds. Moreover, LDH leakage assay was used to confirm the ell killing effect of compound **4** against HCT-116 cells. Treatment with compound **4** (10 µM) for 72 h significantly increased LDH leakage from HCT-116 cells by 2-fold compared to the control cells (Figure 5). On the other hand, compounds **1** and **3** did not induce any significant cell membrane damage in HCT-116 cells at 10 µM concentration after 72 h exposure. Thus, compound **4** possesses cytotoxic properties while compounds **1** and **3** possess antiproleferative effects, which could be attributed to cytostatic effects. However, further biochemical and molecular biology-related experiments are currently underway to define the mechanism of action of compounds **1**, **3** and **4** as cytostatic or cytotoxic agents.

## 3. Experimental Section

### 3.1. General Procedures

Optical rotation was measured on a 241 polarimeter (Perkin Elmer, MA, USA). UV spectra were measured on a Hitachi 300 Spectrophotometer (Hitachi High-Technologies Corporation, Kyoto, Japan). High-resolution ESIMS data were recorded with an Ultra-High Resolution (UHR) TOF spectrometer (Impact, Bruker, Bremen, Germany). NMR spectra were obtained in CDCl_3_ on a Bruker Avance DRX 600-MHz spectrometer at 600-MHz for ^1^H-NMR and 150 MHz for ^13^C-NMR. NMR chemical shifts were expressed in parts per million (ppm) referenced to residual CDCl_3_ solvent signals (δ_H_ 7.26 for ^1^H and δ_C_ 77.0 for ^13^C). Precoated SiO_2_ 60 F_254_ plates (Merck, Darmstadt, Germany) were used for TLC. For column chromatography, SiO_2_ (70–230 mesh, Merck) was used. HPLC purifications were performed on HPLC column (5 µm ZORBAX Eclipse XDB-C18, 250 mm × 4.6 mm, Agilent, CA, USA).

### 3.2. Biological Materials

**Figure 6:**
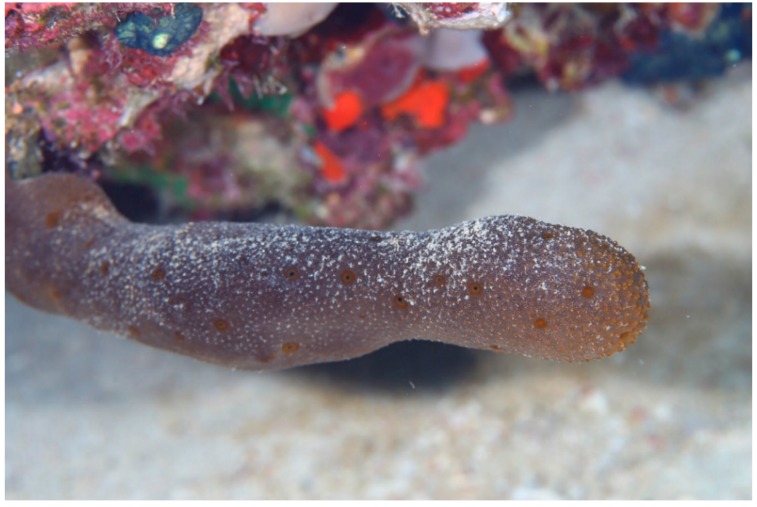
Underwater photograph of the Red Sea sponge *Hyrtios erectus*.

The marine sponge, *Hyrtios erectus* (Keller, 1889) (Figure 6) was collected off Sharm el-Sheikh, Red Sea, Egypt, using scuba diving at a depth of 11 m.

The collected material was immediately frozen and kept at −15 °C until investigation. The sponge was identified by Dr. R. van Soest (Institute of Systematic Population Biology, Amsterdam University, Amsterdam, The Netherlands) to be *Hyrtios erectus* (class Demospongiae, order Dictyoceratida, family Thorectidae) under the number ZMAPOR19761. A voucher specimen has been deposited in the Red Sea invertebrate’s collection at the Faculty of Pharmacy, Suez Canal University, under the registration number SAA-59.

### 3.3. Purification of Compounds ***1**–**4***

The frozen sponge material (900 g, wet wt.) was thawed and extracted at room temperature with MeOH (3 × 2 L). The successive extracts were combined and evaporated under reduced pressure to afford a crude extract (85 gm) which was fractionated on a silica gel column using vacuum liquid chromatography (VLC) with gradient elution (*n*-hexane–CHCl_3_–MeOH) to yield nine fractions (Fr.1 to Fr.9). Fr.4 (2 g) which was eluted with 25% *n*-hexane in CHCl_3_ was fractionated on silica gel column using *n*-hexane–CHCl_3_–MeOH gradient eluent, affording eight subfractions (Fr.4-1 to Fr.4-8).

Fr.4-5 (136 mg) was further subjected to silica gel column chromatography (CC) eluted with *n*-hexane/CHCl_3_ gradient to give nine subfractions (Fr.4-5-1 to Fr.4-5-9). Fr.4-5-6 (34.3 mg) was purified on HPLC (XDB-C18 Zorbax, 5 µm, 250 mm × 4.6 mm) using 90% CH_3_CN/H_2_O at a flow rate of 1.5 mL/min and UV detection at 220 nm to yield compound **1** (1.8 mg) and **2** (1.3 mg).

Fr.4-3 (700 mg) was further subjected to silica gel column chromatography (CC) eluted with *n*-hexane/CHCl_3_ gradient to give 6 subfractions (Fr.4-3-1 to Fr.4-3-6). Fr.4-3-5 (31 mg) was purified on HPLC (XDB-C18 Zorbax, 5 µm, 250 mm × 4.6 mm) using 80% CH_3_CN/H_2_O at a flow rate of 1.5 mL/min and UV detection at 220 nm to yield compound **3** (3.5 mg) and **4** (2.3 mg).

### 3.4. Characterization of 24-Methoxy-petrosaspongia C *(**1**)*

Yellow amorphous solid (1.8 mg); ${\left[\alpha \right]}_{D}^{25}$ +8.7 (*c* 0.15, CHCl_3_); UV (λ_max_, MeOH) (log ε): 226 (4.31), 285 (2.54) nm; NMR data: see Table 1; ESI-MS: *m*/*z* 488.3 [M + H]^+^. HRESIMS: *m*/*z* 488.3374 (calculated for C_29_H_46_NO_5_ [M + H]^+^, 488.3376).

### 3.5. Biological Activity of Compounds ***1**–**4***

The effects of the compounds **1**–**4** on breast adenocarcinoma cells (MCF-7), hepatocellular carcinoma cells (HepG2) and colorectal carcinoma cells (HCT-116) were evaluated using the sulforhodamine B (SRB) assay as previously described [34].

#### 3.5.1. Cell Culture

Breast adenocarcinoma cells (MCF-7), hepatocellular carcinoma cells (HepG2) and colorectal carcinoma cells (HCT-116) were obtained from the National Cancer Institute of Egypt (Giza, Egypt). Cells were maintained in RPMI-1640 supplemented with 100 mg/mL streptomycin, 100 units/mL penicillin and 10% heat-inactivated fetal bovine serum in a humidified, 5% (*v*/*v*) CO_2_ atmosphere at 37 °C. Exponentially growing cells were collected using 0.25% Trypsin-EDTA and plated in 96-well plates at 1000–2000 cells/well. Cells were exposed to serial concentrations of test compounds for 72 h and subsequently fixed with TCA (10%) for 1 h at 4 °C. After several washings, cells were exposed to 0.4% SRB solution for 10 min in dark place and subsequently washed with 1% glacial acetic acid. After drying overnight, Tris-HCl was used to dissolve the SRB-stained cells and color intensity was measured at 540 nm. Doxorubicin was used as a positive control. The dose response curve of compounds was analyzed using a logarithmic best fit equation (E_max_ model Equation).

$$\%\;\text{Cell viability}=\left(100-R\right)\times \left(1-\frac{{\left[D\right]}^{m}}{{K_{d}}^{m}+{\left[D\right]}^{m}}\right)+R$$

where (R) is the residual unaffected fraction (the resistance fraction), (D) is the drug concentration used, (K_d_) is the drug concentration that produces a 50% reduction of the maximum inhibition rate and m is a Hill-type coefficient. IC_50_ was defined as the drug concentration required to reduce absorbance to 50% of that of the control (*i.e.*, K_d_ = IC_50_ when R = 0 and E_max_ = 100 − R) [35].

#### 3.5.2. Cell Membrane Integrity Assessment

The influence of compounds **1**, **3** and **4** on cell membrane integrity was assessed in colorectal adenocarcinoma cells (HCT-116) by LDH leakage assay [36,37]. Briefly, exponentially growing cells were plated in 24-well plates (1 × 10^4^ cells/well). Cells were exposed to 10 µM of tested compounds and compared to untreated cells (control) for 72 h. LDH concentrations were determined in each well using a colorimetric assay (Biosystems, Barcelona, Spain).

## 4. Conclusions

The investigation of the antiproliferative lipophilic extract of the Red Sea sponge *H. erectus* yielded the new metabolite 24-methoxypetrosaspongia C (**1**), along with sesterstatin 3 (**2**), 12-deacetyl-12-*epi*-scalaradial (**3**) and 12-deacetyl-12,18-di-*epi*-scalaradial (**4**). The structures of the isolated compounds were determined by HRESIMS, 1D and 2D NMR data, as well as by comparison with the available data in the literature. The compounds displayed variable growth inhibitory activity against hepatocellular carcinoma cells (HepG2), colorectal carcinoma cells (HCT-116) and breast adenocarcinoma cells (MCF-7) using SRB assay.

## Supplementary Materials

Supplementary materials can be accessed at: http://www.mdpi.com/1420-3049/21/1/82/s1.
