# Supplementary material for: A New Bioactive Metabolite Isolated from the Red Sea Marine Sponge Hyrtios erectus
Source: Molecules. 2016 Jan 15;21(1):82. doi: 10.3390/molecules21010082 (PMC6273094; doi:10.3390/molecules21010082)
Supplement: Supplementary file 1 [file molecules-21-00082-s001.pdf]

Supplementary Materials: A New Bioactive Metabolite Isolated from the Red Sea Marine Sponge *Hyrtios erectus*

Sameh S. Elhady [1,2], Ali M. El-Halawany [2,3], Abdulrahman M. Alahdald [4], Hashim A. Hassanean [1] and Safwat A. Ahmed [1,*]

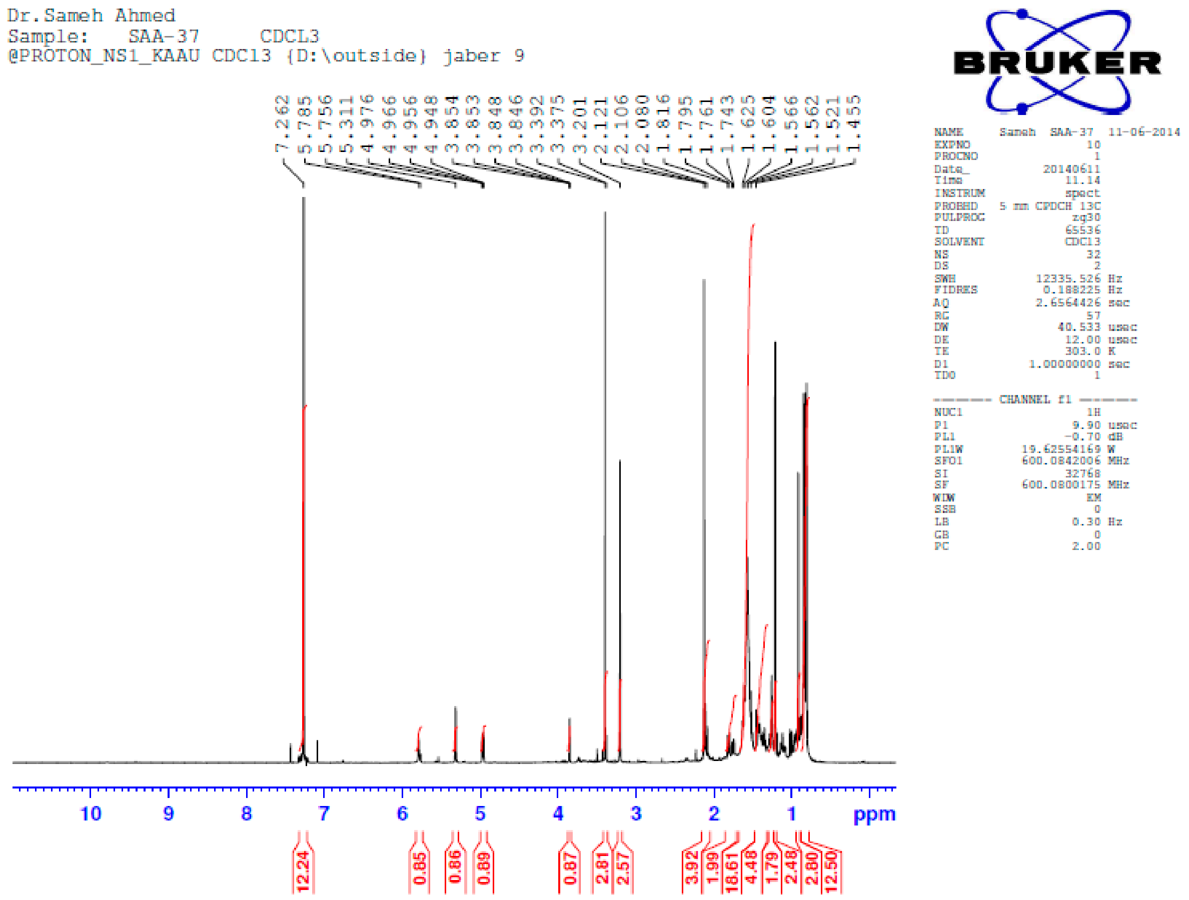

Figure S1. ^{1}H-NMR spectrum of compound **1** ($CDCl_3$).

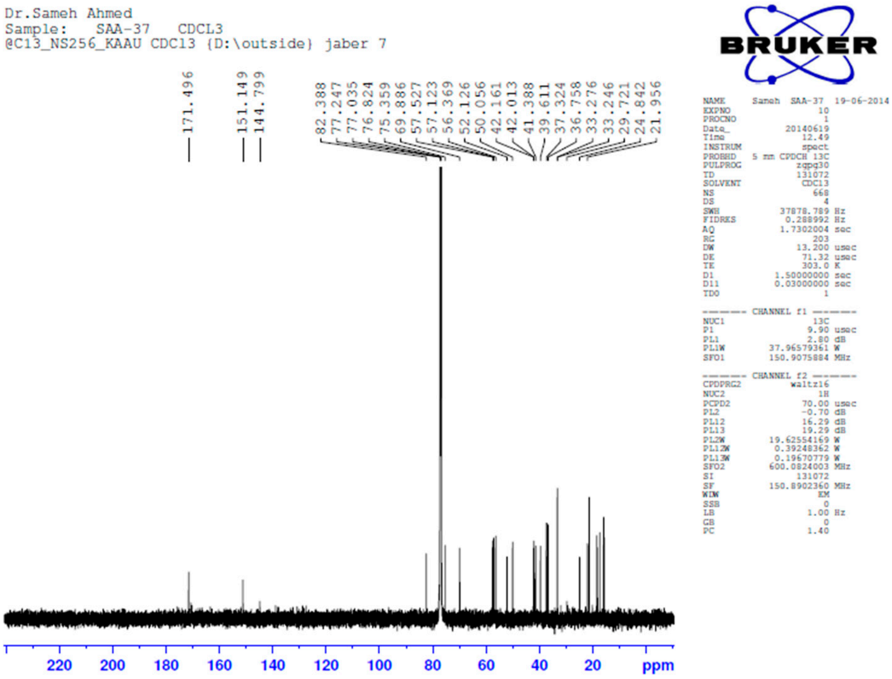

Figure S2. ^{13}C-NMR spectrum of compound **1** ($CDCl_3$).

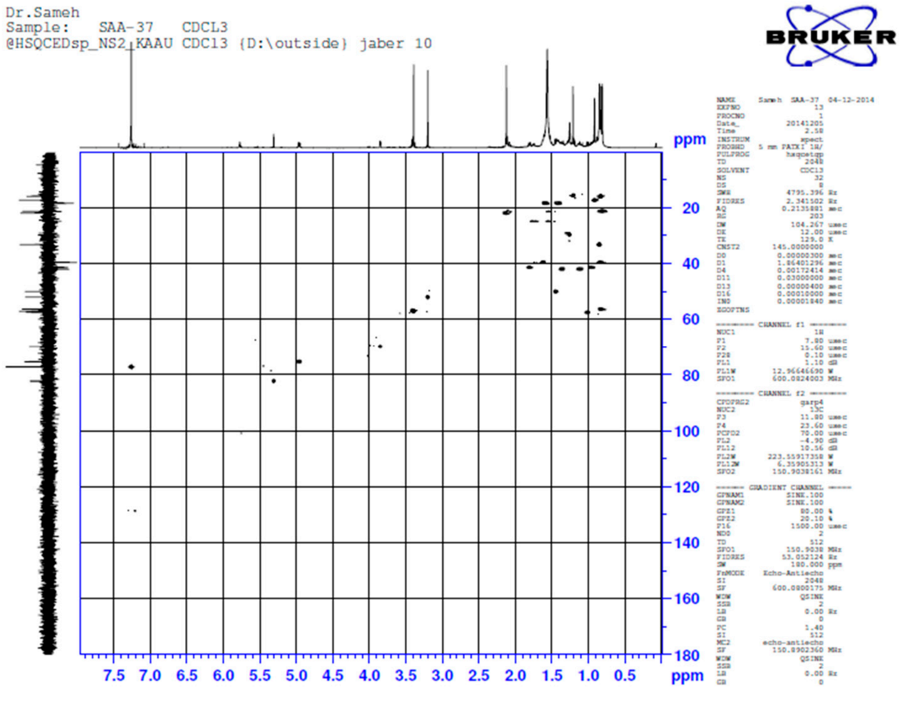

Figure S3. HSQC spectrum of compound **1** ($CDCl_3$).

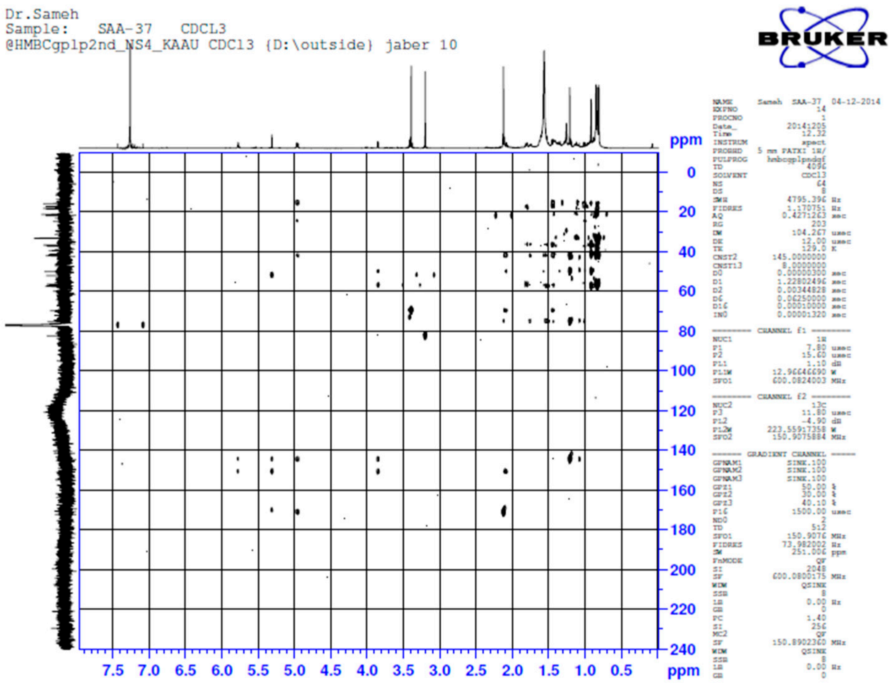

Figure S4. HMBC spectrum of compound **1** ($CDCl_3$).

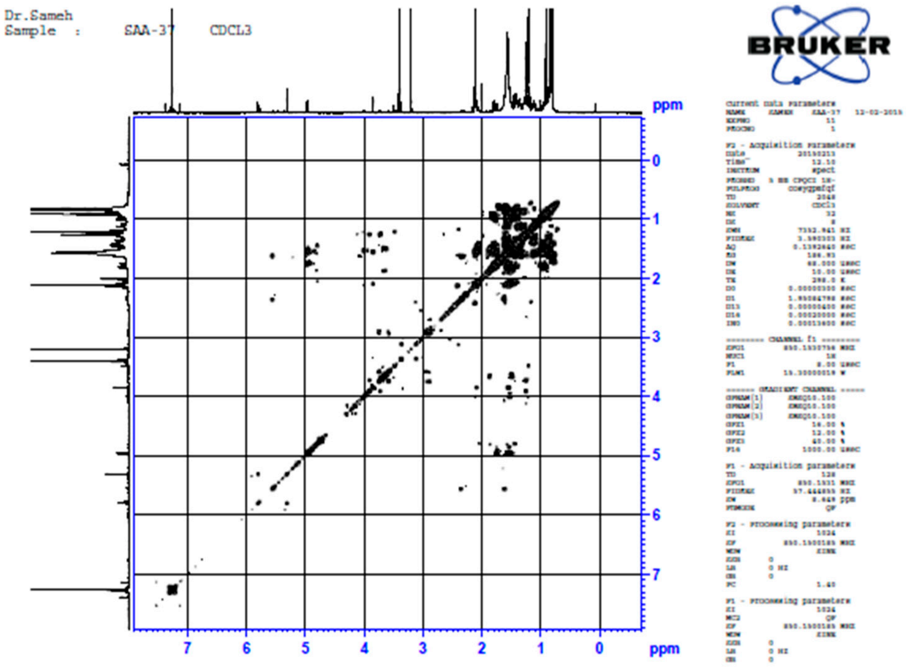

Figure S5. ^{1}H-^{1}H COSY spectrum of compound **1** ($CDCl_3$).

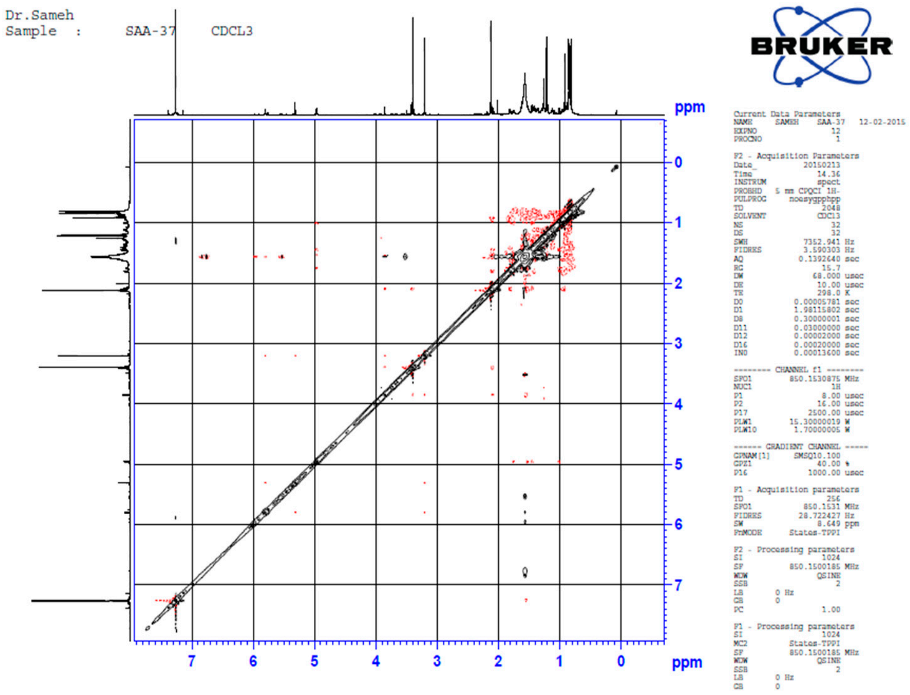

Figure S6. NOESY spectrum of compound **1** ($CDCl_3$).
